# Study on Formation of Phosvitin–Fructooligosaccharide Complex and Stability Improvement Mechanisms

**DOI:** 10.3390/biom16060829

**Published:** 2026-06-03

**Authors:** Anjia Huang, Jingyi Zhang, Shujie Chen, Yinlong Lian, Juan Chen, Xue Zhao, Chenggang Cai

**Affiliations:** School of Biological and Chemical Engineering, Zhejiang University of Science and Technology, Hangzhou 310023, China; 212403817032@zust.edu.cn (A.H.); 222403857027@zust.edu.cn (J.Z.); 212403817018@zust.edu.cn (S.C.); 222503860032@zust.edu.cn (Y.L.); 222503860009@zust.edu.cn (J.C.); 222503855005@zust.edu.cn (X.Z.)

**Keywords:** phosvitin, fructooligosaccharides, non-covalent interaction, stability, molecular docking

## Abstract

Phosvitin (PV) is a highly phosphorylated protein with strong metal-chelating capacity and bioactivity, but its application is hindered by poor environmental stability. In this study, fructooligosaccharides (FOS) were introduced to form a PV–FOS composite system. The interaction mechanism and structural–functional changes were assessed via thermal and pH stability tests, molecular docking, FTIR, circular dichroism (CD), particle size, zeta potential and changes in turbidity. SDS-PAGE and changes in solubility confirmed the coexistence of PV and FOS in the composite system. Molecular docking revealed that FOS with varying degrees of polymerization can bind to PV through non-covalent interactions to form a complex. FTIR showed characteristic peaks of both components, with shifts and intensity changes at 3200–3500 cm^−1^, ~1650 cm^−1^, and 1200–900 cm^−1^, indicating that non-covalent interactions, intermolecular forces that may be hydrogen bonds, occur between amide, carbonyl, and phosphate groups of PV and hydroxyl groups of FOS. CD demonstrated slight secondary structure rearrangement of PV without significant denaturation. Compared with PV alone, the PV-FOS complex showed an increased particle size and a weakly negative surface charge, which could be attributed to the presence of FOS. These changes may enhance the anti-aggregation capacity of the complex. Consistently, turbidity measurements further demonstrated that the PV-FOS complex exhibited better turbidity stability. Functionally, FOS incorporation significantly improved PV’s solubility, metal-chelating capacity, and lipid antioxidant activity under various temperature and pH conditions. In summary, FOS effectively complexes with PV via non-covalent interactions, thereby enhancing structural stability and functionality.

## 1. Introduction

**Figure 1:**
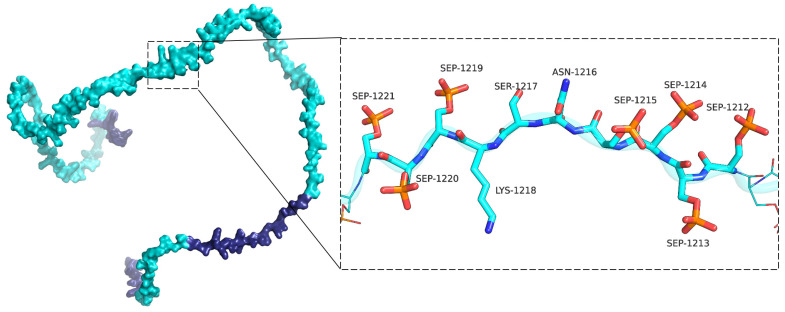
Simulated structure of PV. SEP: phosphorylated serine residues; SER: serine; ASN: asparagine; LYS: lysine.

Egg yolk is rich in unsaturated fatty acids, proteins, vitamins, and minerals. Phosvitin (PV), derived from vitellogenin cleavage, accounts for ~11% of yolk proteins and 4% of yolk dry matter [1,2]. About 50% of its amino acids are phosphorylated serine residues [3], endowing PV with a strong negative charge that prevents a compact hydrophobic core, resulting in a flexible, extended conformation [2,4] (Figure 1). This unique structure grants PV metal-chelating, antioxidant, emulsifying, antimicrobial, and biomineralization-regulating properties [5,6]. For instance, the PV–Fe complex acts as a natural antioxidant and photoprotectant [2,7,8]. Moreover, PV possesses both lipophilic and hydrophilic characteristics, allowing it to adsorb at oil–water interfaces and stabilize emulsions [5,9,10]. Thus, PV is a promising natural antioxidant and emulsifier for functional foods [9,11]. Current preparation methods include ultrasound-assisted thermal extraction [5], simultaneous IgY and PV separation [6], salting-out [11], and chromatography [12,13], laying a foundation for further applications.

Although phosvitin (PV) has some intrinsic stability, its biological activity declines above 65 °C, with partial molecular degradation, limiting its thermal stability [2,14,15]. PV stability is also pH-dependent; its isoelectric point is ~pH 4.0. PV remains relatively stable under neutral or weakly alkaline conditions but is less stable in more acidic environments [11].

**Figure 2:**
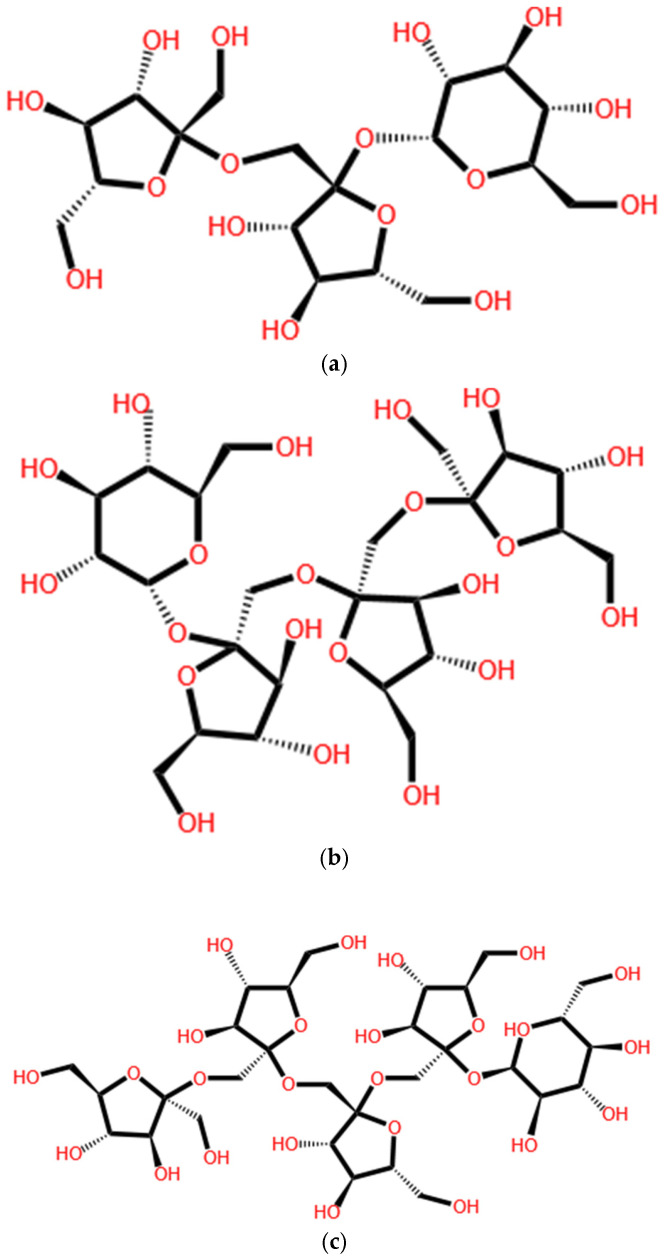
The chemical structure of (**a**) 1-kestose, (**b**) nystose, and (**c**) 1f-fructofuranosylnystose.

Fructooligosaccharides (FOS) are a class of oligosaccharides with sucrose as the basic backbone, in which multiple fructose residues are linked by β-(2→1) glycosidic bonds, with a glucose unit attached at the terminal end of the fructan chain. Typical short-chain FOS mainly include 1-kestose, nystose and 1f-fructofuranosylnystose (Figure 2) [16,17]. They are water-soluble, processing-adaptable, and serve as low-calorie sweeteners and soluble dietary fiber enhancers [16,18]. FOS modulate gut microecology, regulate microbiota structure [17,19], and participate in intestinal barrier maintenance, inflammation regulation, and metabolic regulation [17,19,20]. They also improve the bioavailability of minerals such as Fe^2+^, Ca^2+^, and Mg^2+^ [21,22]. FOS maintain good structural integrity under relatively high temperatures, especially in neutral systems [23,24]; at room temperature, they are stable under neutral and weakly acidic conditions, but stability decreases under extreme pH [24,25,26].

This study introduces FOS to investigate how it modulates the structural and functional properties of PV and the associated mechanisms, offering a foundation for the future development of composite food ingredients.

## 2. Materials and Methods

### 2.1. Materials

Eggs were purchased from a local market in Hangzhou, China. Ferrozine was obtained from Aladdin Corp. Ltd. (Shanghai, China). Fructooligosaccharides (FOS), Tris, glycine, MES, sodium acetate, and ferrous sulfate (analytical grade) were purchased from Macklin Corp. Ltd. (Shanghai, China). The FOS used is a mixture of 1-kestose (GF2), nystose (GF3), and 1f-fructofuranosylnystose (GF4), with FOS purity > 90%, and total content of glucose, sucrose and fructose <10%. Phosphate-buffered saline (PBS) was purchased from Servicebio (Wuhan, China). The BCA total protein assay kit and the MDA assay kit were purchased from Nanjing Jiancheng Bioengineering Institute (Nanjing, China).

### 2.2. Preparation of PV-FOS Complex Solution

PV was extracted following the methods of Jiang et al. [27] and Marcet et al. [11]. FOS was dissolved in 10 mM PBS (pH 7.4) at concentrations of 3%, 5%, 7%, 9%, and 11% (*w*/*v*). PV was prepared at 10 mg/mL in the same buffer. The FOS solution was added dropwise to the PV solution at a 1:1 volume ratio under stirring, and the mixture was stirred for 2 h to obtain the complex solution. A PV solution (5 mg/mL) served as the control. The protein solubility of the supernatant was then measured.

### 2.3. Stability Analysis

For thermal solubility assays, 10 mM PBS (pH 7.4) was used. For metal chelating and lipid antioxidant capacity assays, 10 mM Tris-HCl (pH 7.4) was used instead of PBS to avoid interference from phosphate ions. The prepared PV and PV–FOS solutions were incubated at 25, 40, 60, 80, 100, and 121 °C.

The following buffers (10 mM each) were used for pH stability analysis: glycine-HCl (pH 2.0), sodium acetate-acetic acid (pH 4.0), MES (pH 6.0), Tris-HCl (pH 8.0), glycine-NaOH (pH 10.0), and KCl-NaOH (pH 12.0). After dissolving and incubating the samples at room temperature for 1 h, the pH of each solution was adjusted to 7.4 using 1 M HCl or 1 M NaOH, and the volume added was recorded.

### 2.4. Molecular Docking Simulation

Molecular docking simulations were performed using AutoDockTools-1.5.7 and PyMOL software (version 3.03). FOS with three degrees of polymerization, namely 1-kestose (GF2), nystose (GF3), and 1-fructosylnystose (GF4), were docked with PV.

### 2.5. Structural Characterization of PV-FOS Complex

#### 2.5.1. Molecular Weight and Purity Analysis

PV was analyzed by SDS-PAGE [28]. Solid samples were dissolved to ~2 mg/mL. Then, 40 μL of sample solution was mixed with 10 μL of 5× SDS-PAGE loading buffer (type PH0333, Fuzhou Feijing Biotechnology Co., Ltd, Fuzhou, China) and heated in boiling water for 10 min. After electrophoresis, the gel was stained for 2 h at room temperature, then destained with solution changes every 1 h until the background was light. The electrophoretogram was analyzed using SHST Capture Gel software (Shenhua Science Technology Co., Ltd. Hangzhou, China, version 2.0.0.10). Relative molecular weights were calculated using a pre-stained protein marker (10–170 kDa), and purity was determined by grayscale integration.

#### 2.5.2. Functional Group Analysis

A mixture of 2 mg of PV or PV–FOS complex and 200 mg of KBr was ground and pressed into a pellet. Infrared spectra were recorded using a Vertex 70 spectrometer (Bruker Ettlingen, Germany) over 500–4000 cm^−1^ (absorption mode, 32 scans, resolution 4 cm^−1^).

#### 2.5.3. Secondary Structure Analysis

Far-UV circular dichroism (CD) spectra were recorded from 200 to 500 nm using a MOS-450 CD spectrometer (Bio-Logic, Claix, France). PV and PV–FOS concentrations were 0.3 mg/mL. PV and PV-FOS was dissolved in ultrapure water. Solvent background spectra were subtracted. Secondary structure was analyzed using CDpro software (version 2.1.0.223).

#### 2.5.4. Average Particle Size and ζ-Potential Analysis of the Complex

The average particle size and ζ-potential of PV and PV–FOS complexes were evaluated using a Zetasizer Nano-ZS (Malvern, Worcestershire, UK). Sample solutions were diluted to 2 mg/mL. All experiments were performed in triplicate.

### 2.6. Functional Characterization of PV-FOS Complex

#### 2.6.1. PV-FOS Complex Solubility

The solubility of PV was determined by measuring the total protein content in the supernatant of PV and PV-FOS solutions using the BCA method. Under alkaline conditions, proteins reduce Cu^2+^ to Cu^+^, which forms a purple complex with the BCA reagent exhibiting maximum absorbance at 562 nm. Since absorbance is proportional to protein concentration, the protein concentration was calculated accordingly.

#### 2.6.2. PV-FOS Complex Metal Ion Chelating Capacity

According to the ferrozine assay described by Lawrence [29], in which ferrozine chelates Fe^2+^ to form a purple-red complex, the metal-chelating capacity of PV and PV-FOS was determined as follows: A 5 mM ferrozine solution was prepared in ultrapure water and stored in the dark. A 1 mM FeSO_4_ solution was prepared in 0.1 M HCl and also stored in the dark. In an Eppendorf tube, 50 μL of sample (PV or PV-FOS) was mixed with 50 μL of FeSO_4_ solution and 800 μL of ultrapure water. After thorough mixing, the mixture was incubated for 30 min to allow saturated chelation of Fe^2+^ by the sample. Subsequently, 100 μL of ferrozine solution was added, mixed well, and reacted for another 30 min for full color development. Absorbance was measured at 562 nm. A lower absorbance indicated a higher amount of Fe^2+^ chelated by the sample. For the standard curve, FeSO_4_ solutions at concentrations of 0, 0.0625, 0.125, 0.25, 0.5, 1.0, and 2.0 mM were prepared in 0.1 M HCl. For each concentration, 50 μL was transferred to an Eppendorf tube, mixed with 100 μL of ferrozine solution and 850 μL of ultrapure water, incubated at room temperature for 30 min, and the absorbance was measured at 562 nm.

#### 2.6.3. PV-FOS Complex Lipid Antioxidant Capacity Analysis

According to Wang et al. [30], egg yolk lecithin oxidizes faster than soybean lecithin; therefore, egg yolk lecithin was chosen as the oxidation substrate. Following the method of Ishikawa et al. [8], the procedure for evaluating the lipid antioxidant capacity of PV and PV-FOS was designed as follows: A 5 mM FeSO_4_ solution was prepared in 0.1 M HCl and stored in the dark. Egg yolk lecithin was dissolved in ultrapure water at a concentration of 10 mg/mL, sonicated in an ice bath to form liposomes, and stored in the dark. In an Eppendorf tube, 100 μL of the lecithin liposome solution was mixed with 100 μL of PV or PV-FOS solution, and finally 25 μL of FeSO_4_ solution was added to initiate the reaction. After incubation at room temperature under natural light for 1 h, the MDA content in the tube was measured. A lower MDA content indicated a stronger lipid antioxidant capacity.

## 3. Results and Analysis

### 3.1. Preparation of FOS-PV Complex

**Figure 3:**
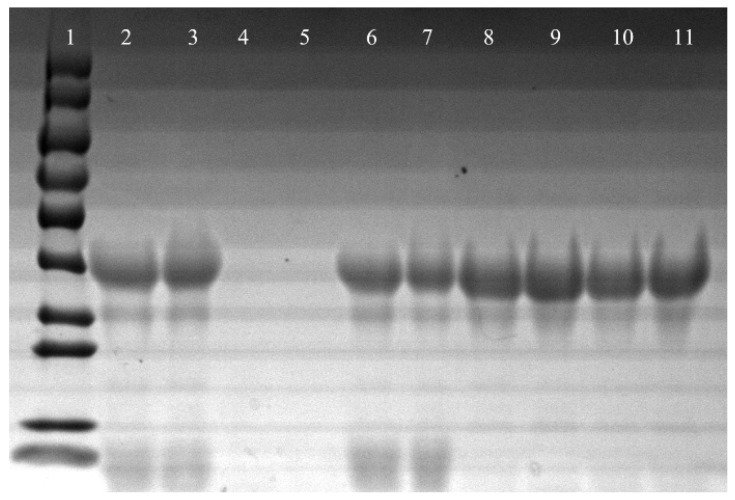
SDS-PAGE of PV and PV-FOS. Lane 1: marker; Lane 2: supernatant of PV solution treated at 25 °C; Lane 3: supernatant of PV solution treated at 70 °C; Lanes 4 and 5: FOS solution samples treated at 25 °C and 70 °C, respectively; Lane 6: PV-5% FOS mixed solution treated at 25 °C; Lanes 7–11: supernatants of PV-FOS solutions (with 3%, 5%, 7%, 9%, and 11% FOS) treated at 70 °C.

**Figure 4:**
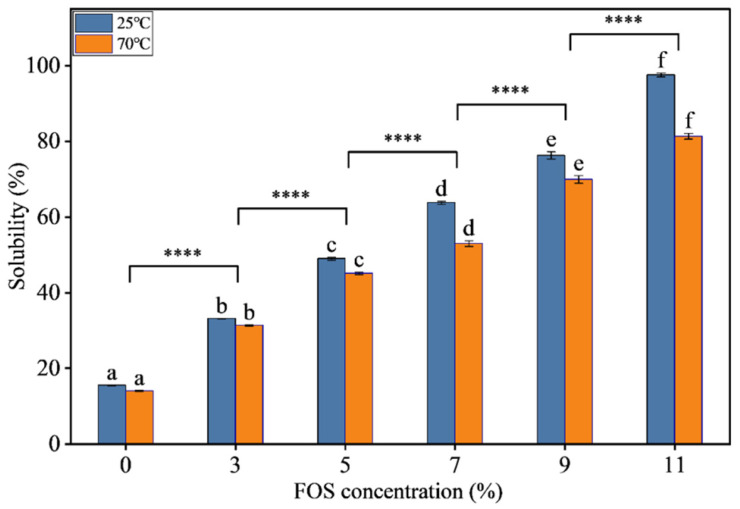
Influence of FOS concentration on PV solutility. Error bars represents average of three different results. **** indicates *p* < 0.0001. Different lowercase letters indicate statistically significant differences between groups (one-way ANOVA followed by Tukey’s multiple comparison test, *p* < 0.05), while the same letter indicates no significant difference (*p* > 0.05). Data are presented as mean ± standard deviation, *n* = 3.

PV was extracted and prepared according to the method in Section 2.2; the molecular weight analysis was performed by SDA-PAGE (Figure 3). The mixed reaction of FOS solution and PV solution with different concentrations was carried out. For the supernatant after centrifugation, the solubility of PV increased with increasing FOS concentration (Figure 4). Based on these results, an FOS concentration of 10% (*w*/*v*) was selected for subsequent experiments.

Based on SDS-PAGE analysis of the supernatant and quantitative solubility measurements, the soluble protein content increased progressively with rising FOS concentration, and distinct PV bands were consistently observed across all samples. These results demonstrate that PV remains soluble and molecularly dispersed in the presence of FOS, indicating that FOS enhances both the apparent solubility and colloidal stability of PV.

### 3.2. Formation Mechanism and Characteristics of PV-FOS Complex

To investigated the formation mechanism of the PV-FOS complex, molecular docking, FTIR, CD, particle size and ζ-potential analysis were conducted.

#### 3.2.1. Visualization of Docking Function

**Figure 5:**
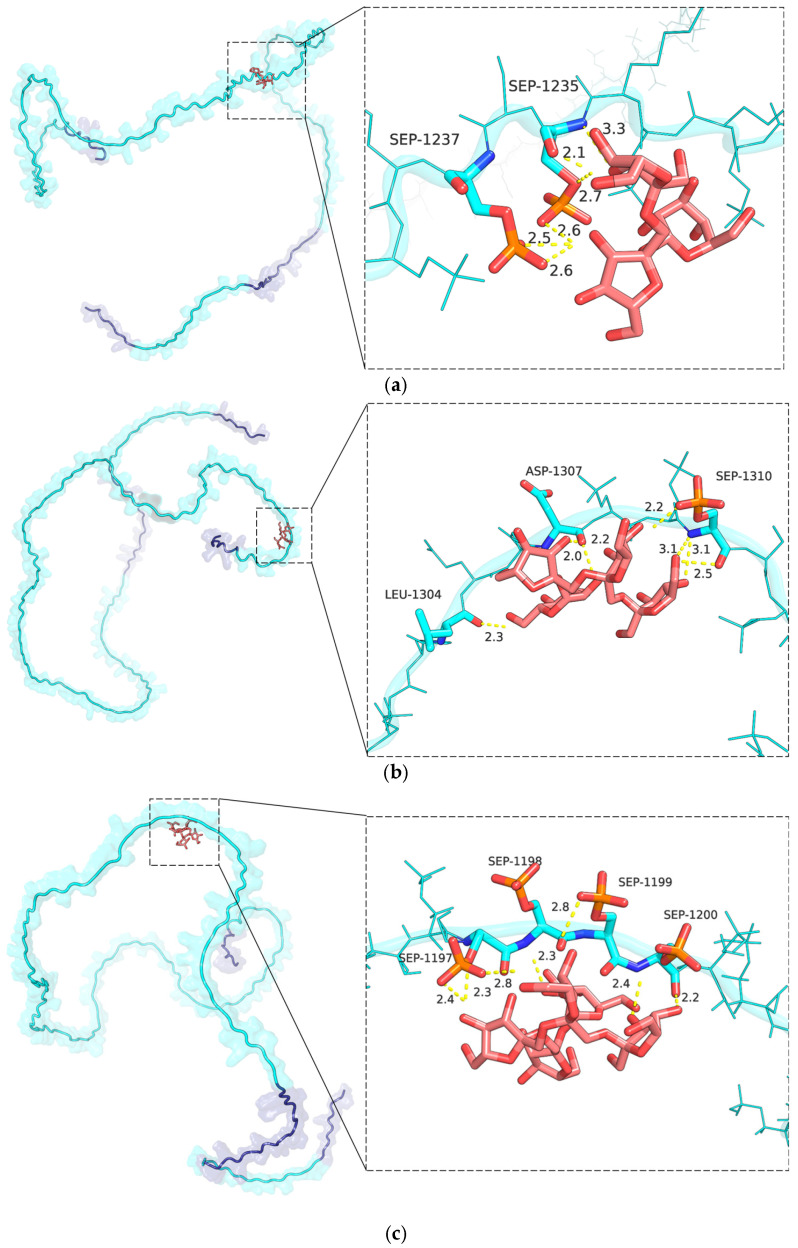
Docking diagrams of (**a**) 1-kestose with PV, (**b**) nystose with PV, and (**c**) 1f-fructofuranosylnystose with PV.

1-kestose (GF2), nystose (GF3), and 1f-fructofuranosylnystose (GF4) potentially interact with PV through non-covalent bonds (Figure 5). Its predicted binding sites were mainly located near the surface-exposed phosphorylated serine-rich regions of PV, with interaction distances of 2.1–3.3 Å, which fall within the typical range for hydrogen-bonding polar contacts. This suggests that hydrogen-bonding polar interactions may contribute to the interaction between PV and FOS. These interactions may involve the hydroxyl groups of FOS and the phosphate groups or other polar residues of PV. However, molecular docking provides only a static prediction; therefore, the specific binding sites and binding forces require further investigation.

#### 3.2.2. Functional Group Analysis Results

**Figure 6:**
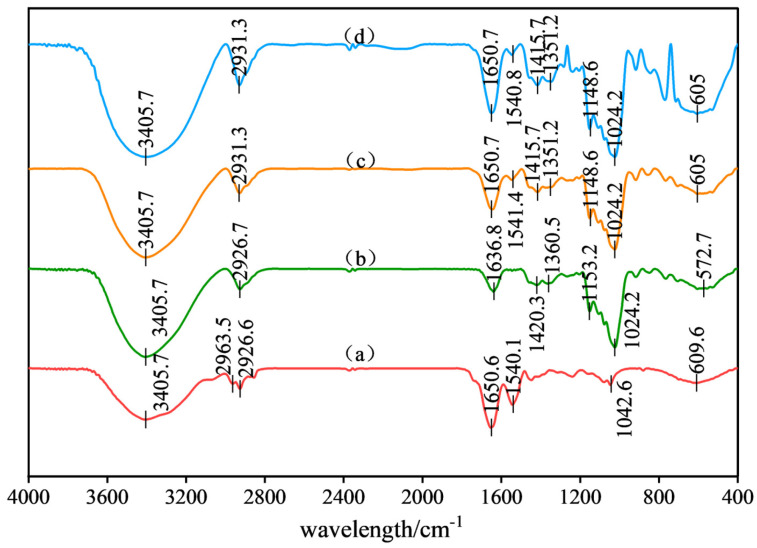
FTIR analysis of (a) PV, (b) FOS, (c) PV-FOS after ultrafiltration to remove unbound FOS, and (d) PV-FOS without ultrafiltration treatment.

The functional groups of PV, FOS, and their complex were analyzed by Fourier transform infrared spectroscopy (Figure 6). (a) represents PV, which shows a typical amide I band at approximately 1650 cm^−1^, an amide II band at 1540 cm^−1^, and an absorption peak around 1040 cm^−1^ corresponding to the symmetric/asymmetric stretching vibration peak of phosphate groups. (b) represents FOS, which exhibits a flat profile at about 1540 cm^−1^, and the strong absorption peaks appearing in the range of 1200–900 cm^−1^ are mainly associated with C-O, C-O-C and glycosidic bonds.

Compared with the single component, the FTIR spectrum of the PV-FOS complex retains the main characteristic absorption peaks of both PV and FOS, proving that both substances exist in the complex. At the absorption peak around 1650 cm^−1^, a slight shift occurs in the amide I band of FOS. For the absorption peak at approximately 1540 cm^−1^, the complex maintains the amide II band of PV relative to FOS. In the range of 1200–900 cm^−1^, the complex still exhibits distinct absorption bands; however, compared with pure FOS, both its absorption intensity and peak shape have changed.

The changes in the FTIR spectra suggested alterations in the microenvironments of hydroxyl, amide, carbonyl, and phosphate groups, providing evidence for non-covalent interactions between molecules between PV and FOS.

#### 3.2.3. Analysis of PV Secondary Structure

**Figure 7:**
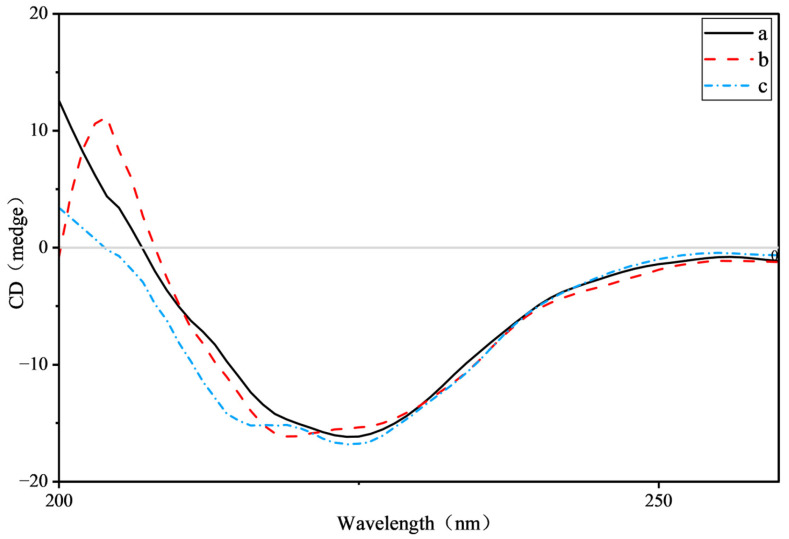
CD spectroscopic analysis of (a) PV, (b) PV-FOS after ultrafiltration to remove unbound FOS, and (c) PV-FOS without ultrafiltration treatment.

The circular dichroism (CD) results showed that PV exhibited a distinct negative band in the region of approximately 220–225 nm, indicating that its secondary structure is not predominantly composed of a typical α-helical conformation (Figure 7). Compared with pure PV, both ultrafiltered and non-ultrafiltered PV-FOS exhibited certain changes in CD signals, while all of them retained the absorption peak signals of the secondary structure of PV (Table 1). This further confirms that there is an interaction between PV and FOS, and FOS can induce a slight rearrangement of the secondary structure of PV.

#### 3.2.4. Physicochemical Properties of Colloids

**Figure 8:**
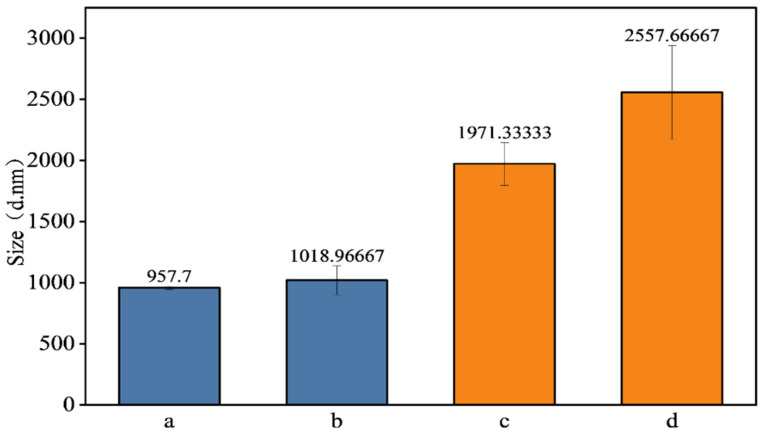
Particle size analysis of (a) PV, (b) FOS, (c) PV-FOS after ultrafiltration (unbound FOS removed), and (d) PV-FOS without ultrafiltration treatment.

**Figure 9:**
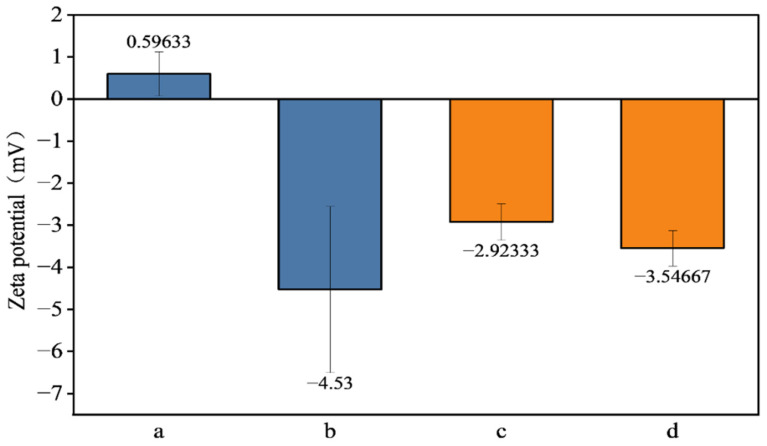
ζ-Potential analysis of (a) PV, (b) FOS, (c) PV-FOS after ultrafiltration (unbound FOS removed), and (d) PV-FOS without ultrafiltration treatment.

**Figure 10:**
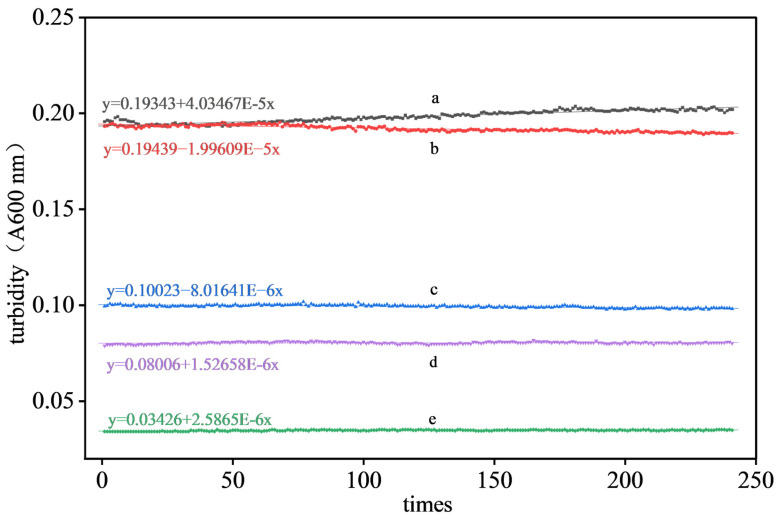
Changes in turbidity of (a) PV, (b) PV-FOS, (c) the supernatant of PV-FOS, (d) the supernatant of PV, and (e) FOS.

As shown by the particle size results, the PV-FOS system exhibited a larger particle size than PV or FOS alone (Table 2). In addition, the relatively high PdI value indicated a broad particle size distribution, suggesting that the PV-FOS system formed polydisperse aggregates rather than uniform nanoscale particles (Figure 8). The ζ-potential results showed that the surface charge of the PV-FOS mixture shifted toward more negative values, which may be attributed to the contribution of negatively charged FOS to the particle surface (Figure 9). However, the absolute value of the zeta potential remains relatively low, so the overall stability of the complex may be inferior to that of complexes reported in other studies. This change in surface charge could contribute to the improved dispersion behavior of the system. Consistently, turbidity measurements showed that the turbidity of the PV-FOS system remained relatively stable, whereas PV alone showed a slight increase in turbidity, suggesting a tendency toward partial aggregation (Figure 10). Therefore, FOS incorporation may improve the apparent dispersion stability of PV to some extent. However, considering the relatively low absolute ζ-potential values and the high PdI of the PV-FOS system, this stabilization should be regarded as limited and cannot be simply attributed to strong electrostatic repulsion. Further studies are required to clarify the underlying stabilization mechanism.

### 3.3. Function of PV-FOS Complex

To evaluate the stability of PV and PV-FOS complexes, their thermal and acidic stabilities were investigated.

#### 3.3.1. Thermal Stability of PV and PV-FOS Complex

**Figure 11:**
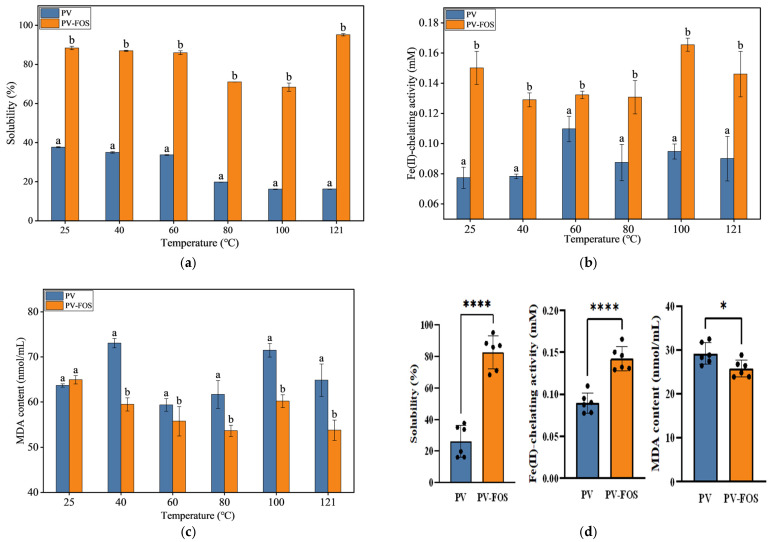
Comparison of stability between PV and PV-FOS under different temperature treatments. Different letters above the bars indicate significant differences (*p* < 0.05). (**a**) Solubility (%); (**b**) Fe(II)-chelating activity (mM); (**c**) MDA content (nmol/mL); (**d**) overall comparison between PV and PV-FOS. * indicate significant differences between groups. * indicates *p* < 0.05, **** indicates *p* < 0.0001.

Under heat treatment at different temperatures, the solubility of PV in the PV-FOS complex was significantly higher than that of PV alone (Figure 11). Within the temperature range of 80 °C to 121 °C, the solubility of PV decreases rapidly, while PV-FOS still maintains a high solubility. This indicates that FOS can inhibit the aggregation and precipitation of PV. At 121 °C in particular, the solubility of PV in the PV-FOS system shows an upward trend. It is speculated that high temperature and high pressure induce the Maillard reaction.

PV-FOS exhibited stronger Fe^2+^-chelating capacity than PV alone after heat treatment at different temperatures; the standard curve with the linear regression equation y = 0.0049 + 0.9793x (R^2^ = 0.99995) was used for Fe^2+^ analysis. At room temperature (25 °C), the metal ion chelating capacity of PV-FOS is significantly higher than that of PV, which may be attributed to the influence of FOS on the secondary structure of PV. The metal ion chelating capacity of PV reaches its maximum at 60 °C, possibly because moderate heating causes partial folding of PV and exposes more sites capable of binding metal ions. With the continuous rise in temperature, PV aggregates and consequently buries its active sites. The metal ion chelating capacity of PV-FOS peaks at 100 °C. This is probably because FOS can induce limited unfolding of PV during heating without triggering aggregation, thereby retaining more accessible metal binding sites even at high temperatures.

At 25 °C, the MDA content produced by the mixed systems added with PV solution and PV-FOS solution is similar. When the temperature rises to 40 °C, it promotes the progress of lipid oxidation reaction and increases the MDA content of the PV solution system. Within the temperature range of 60 °C to 80 °C, moderate heating induces structural rearrangement of PV, exposes active sites and reduces MDA generation. Further temperature elevation destroys the structure of PV and accelerates the lipid oxidation process. Throughout the overall heating trend, the lipid antioxidant capacity of the PV-FOS composite system is superior to that of PV. In conclusion, the PV-FOS composite system possesses good thermal stability.

#### 3.3.2. Acid–Base Stability of PV and PV-FOS Complex

**Figure 12:**
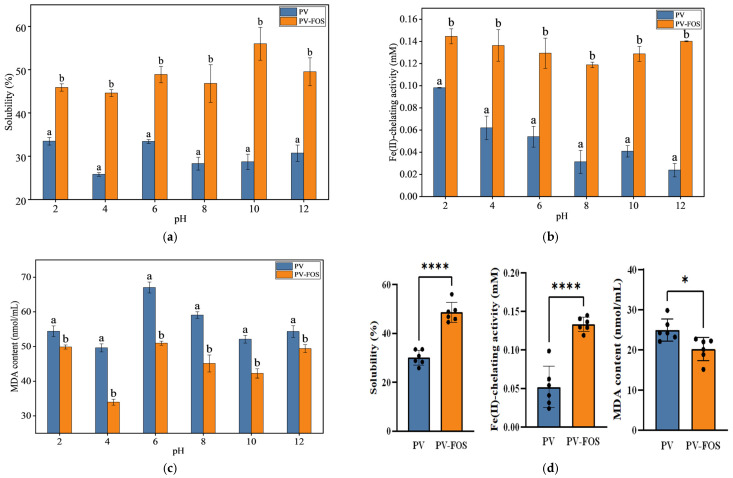
Comparison of stability between PV and PV-FOS under different pH conditions. Bars labeled with different letters denote statistically significant differences (*p* < 0.05). (**a**) Solubility (%); (**b**) Fe(II)-chelating activity (mM); (**c**) MDA content (nmol/mL); (**d**) overall comparison between PV and PV-FOS. * indicates *p* < 0.05, **** indicates *p* < 0.0001.

The solubility of PV-FOS was significantly higher than that of PV alone across all tested pH conditions results (Figure 12). The overall solubility of PV was lower than that of PV-FOS under different pH conditions, indicating that PV tends to undergo molecular aggregation under acidic or alkaline environments. In contrast, PV-FOS consistently maintained a higher solubility, demonstrating that the introduction of FOS significantly improved the dispersion state of the protein across various pH environments. At pH 2, the charge of the system increased and the intermolecular repulsive force was enhanced, resulting in higher solubility of PV and PV-FOS compared with that at pH 4. Since pH 4 is close to the isoelectric point of PV, PV exhibited the lowest solubility at this pH value, while the introduction of FOS enabled it to maintain a relatively high solubility. Consequently, PV-FOS exhibited higher solubility and better system stability under different pH conditions.

For Fe^2+^ concentration analysis, the linear equation formula of y = 0.0049 + 0.9793x (R^2^ = 0.99995) was used. The Fe^2+^-chelating capacity of PV-FOS was consistently higher than that of PV, with relatively little fluctuation across the pH range. This indicates that complexation with FOS improves the metal ion binding stability of phosvitin under acidic and alkaline environments. The ionization state of functional groups on the protein surface is significantly affected by pH, which in turn alters the charge distribution, conformational state, and interaction with metal ions. For PV, pH changes may affect the dissociation degree of phosphate and carboxyl groups, induce conformational adjustments or even aggregation, and thus influence the exposure of metal binding sites. By contrast, PV-FOS maintained a high chelating capacity over a broad pH range, suggesting that hydrogen bonding and hydration protection provided by FOS mitigate the pH-induced structural disruption of PV, thereby preserving its metal binding activity.

The MDA content results further illustrate the impact of pH on functional activity. Compared with PV, PV-FOS exhibited lower MDA levels across the entire pH range, indicating stronger lipid antioxidant activity. When the environment is close to the isoelectric point, i.e., at pH = 4, the PV-FOS mixed system exhibits the lowest MDA production. This may be attributed to the structural changes of PV as pH decreases, which expose active sites and thereby enhance the lipid antioxidant capacity, further reducing MDA formation. In addition, the higher solubility of the composite system indicates better dispersibility and more sufficient exposure of functional sites, which also helps improve antioxidant activity. Therefore, PV-FOS maintains excellent antioxidant properties under acidic, neutral and alkaline conditions and possesses a wide pH adaptation range.

## 4. Discussion

These theoretical findings lay the groundwork for future studies on the application of composite particles and colloidal systems. Such mixtures hold promise as multifunctional food ingredients for the delivery of prebiotic–mineral nutrients.

Recent research indicates that complexing PV with polyphenols (e.g., PV-resveratrol and PV-gallic acid) can further boost its antioxidant potential via free radical scavenging and metal chelation, respectively. Compared with the PV-FOS mixture investigated in this study, the covalent PV–gallic acid complex exhibited greater structural stability and stronger anti-aggregation capacity, although its functional performance was relatively limited. By contrast, the PV–resveratrol complex showed a smaller particle size and a more homogeneous dispersion [27,31]. Compared with the PV-FOS system investigated in this study, the covalent PV–pectin conjugate shows improved emulsifying properties and thermal stability, along with a stronger negative surface charge. This stronger negative charge may enhance electrostatic repulsion between particles, thereby contributing to improved colloidal stability [32]. The covalent PV–galactomannan complex retained favorable antioxidant activity after thermal treatment and exhibited good structural stability. However, compared with the PV-FOS mixture, its preparation required a longer reaction time. This finding provides a useful reference for future studies on the covalent modification of PV-FOS systems. [33]. Additionally, leveraging PV functional synergy with IgY can significantly enhance its antibacterial effect, and findings provide valuable insights for future research on the antibacterial activity of PV and its enzymatic peptides [34,35].

## 5. Conclusions

Collectively, these results suggest that PV and FOS can form a composite system through non-covalent intermolecular interactions. SDS-PAGE and solubility analyses confirmed the coexistence of PV and FOS in the same system and indicated that FOS improved the dispersion state of PV. Molecular docking visualization further showed that FOS with different degrees of polymerization could interact with PV at multiple binding sites, potentially through hydrogen bonding. FTIR analysis revealed changes in the microenvironments of hydroxyl, amide, carbonyl, and phosphate groups, which were consistent with the involvement of intermolecular hydrogen bonds or other non-covalent interactions. Nevertheless, these interactions require further experimental verification. CD spectroscopy indicated slight alterations in the secondary structure of PV, suggesting conformational adjustments during mixture formation. Particle size analysis showed that the PV-FOS system exhibited significantly larger particle sizes than PV or FOS alone, indicating the formation of large particles or aggregates, while the high PdI values reflected the pronounced polydispersity of the system. In addition, ζ-potential and turbidity measurements demonstrated that FOS enhanced the dispersion stability of PV. The surface charge shifted from nearly neutral for PV to weakly negative for the PV-FOS system, which increased the apparent negative charge and may strengthen interparticle repulsion, thereby improving dispersion stability.

Functionally, the PV–FOS complex exhibited enhanced solubility, stronger Fe^2+^-chelating capacity (i.e., metal ion chelating capacity), and lower malondialdehyde (MDA) content (indicating superior lipid antioxidant capacity) across various temperature and pH conditions. These results indicate that the incorporation of FOS significantly improves the functional stability and environmental tolerance of PV. The improved performance may result from the formation of composite aggregates through non-covalent intermolecular interactions between PV and multiple FOS molecules, which enhances the dispersibility of PV. Nevertheless, the current findings cannot rule out the potential contribution of nonspecific aggregation or changes in the solution environment. Overall, this study provides a theoretical basis for the future development of composite food ingredients designed for prebiotic–mineral nutrient delivery.

## Figures and Tables

**Table 1:** Secondary structure proportion of the sample.

Sample	Helix	Sheet	Turns	Unordered
PV	22.22%	20.80%	15.81%	41.10%
PV-FOS complex (ultrafiltration)	21.58%	21.67%	16.93%	39.81%
PV-FOS complex (without ultrafiltration)	24.62%	20.04%	16.30%	39.04%

**Table 2:** PdI analysis of PV, FOS, and their complexes.

Sample	PdI
PV	0.66 ± 0.03
FOS	0.73 ± 0.01
PV-FOS complex (ultrafiltration)	0.92 ± 0.10
PV-FOS complex (without ultrafiltration)	0.93 ± 0.12

## Data Availability

The original contributions presented in this study are included in the article. Further inquiries can be directed to the corresponding author.
